# Chicken faecal microbiota and disturbances induced by single or repeated therapy with tetracycline and streptomycin

**DOI:** 10.1186/1746-6148-9-30

**Published:** 2013-02-13

**Authors:** Petra Videnska, Marcela Faldynova, Helena Juricova, Vladimir Babak, Frantisek Sisak, Hana Havlickova, Ivan Rychlik

**Affiliations:** 1 Veterinary Research Institute, Hudcova 70, 621 00, Brno, Czech Republic

**Keywords:** Chicken, Microbiome, Intestinal tract, Pyrosequencing, Tetracycline, Streptomycin

## Abstract

**Background:**

In this study, we characterised the microbiota present in the faeces of 15- and 46-week-old egg laying hens before and after tetracycline or streptomycin therapy. In the first experiment, the layers were subjected to 7 days of therapy. In the second experiment, the hens were subjected to two days of therapy, which was repeated for an additional two days after 12 days of antibiotic withdrawal. This enabled us to characterise dynamics of the changes after antibiotic administration and withdrawal, and to identify genera repeatedly resistant to tetracycline and streptomycin.

**Results:**

Real-time PCRs specific for *Enterobacteriales*, *Lactobacillales*, *Clostridiales* and *Bifidobacteriales* showed that changes in the microbiota in response to antibiotic therapy and antibiotic withdrawal were quite rapid and could be observed within 24 hours after the change in therapy status. Pyrosequencing of PCR amplified V3/V4 variable regions of 16S rRNA genes showed that representatives of the orders *Clostridiales*, *Lactobacillales*, *Bacteroidales, Bifidobacteriales*, *Enterobacteriales*, *Erysipelotrichales*, *Coriobacteriales*, *Desulfovibrionales*, *Burkholderiales*, *Campylobacterales* and *Actinomycetales* were detected in the faeces of hens prior to the antibiotic therapy. Tetracycline and streptomycin therapies decreased the prevalence of *Bifidobacteriales*, *Bacteroidales*, *Clostridiales*, *Desulfovibrionales*, *Burkholderiales* and *Campylobacterales* in faecal samples in both experiments. On the other hand, *Enterobacteriales* and *Lactobacillales* always increased in prevalence in response to both therapies. Within the latter two orders, *Escherichia* and *Enterococcus* were the genera prevalence of which increased after all the antibiotic treatments.

**Conclusions:**

The changes in microbiota composition induced by the antibiotic therapy were rapid and quite dramatic and only representatives of the genera *Enterococcus* and *Escherichia* increased in response to the therapy with both antibiotics in both experiments.

## Background

The gut microbiology of *Gallus gallus* has received considerable prior attention, however, the majority of experiments have been performed with broilers, and the gut microbiota composition in egg laying hens has been characterised much less frequently [1-4]. The reason why the majority of experiments have been performed in broilers is quite clear, as the gut microbiota, especially its altered development, significantly reduces the profitability of broiler producers [5].

The whole issue of the development of gut microbiota in newly hatched chickens is further complicated by a total absence of hens as donors of healthy microbiota during the hatching of chickens in commercial production. The gut colonisation of chickens in commercial production immediately after hatching is therefore dependent on environmental sources only. The first colonisers usually belong to *Enterobacteriales* followed by representatives of *Clostridiales* and *Lactobacillales*[1,3,6-8]. Our unpublished results show that these initial colonisers are frequently resistant to commonly used antibiotics such as tetracycline, streptomycin or ampicillin. The initial colonisation may therefore lead to the establishment of antibiotic resistant microbiota followed by a prolonged persistence of antibiotic resistant bacteria in the intestinal tract of chickens. However, for how long and how easily such resistant clones can be positively selected for by antibiotic therapy later during chicken life, is relatively unknown. The results obtained from humans or mice indicate that the changes induced by antibiotic therapy are quite severe but relatively soon after withdrawal of the therapy, within 2 weeks, the microbiota composition returns back to the state prior to the therapy [9-12]. In this study we therefore characterised the chicken faecal microbiome and changes induced by antibiotic therapy. The obtained results allowed us to identify bacterial genera present in the chicken gut microbiome, and out of these the genera which were repeatedly resistant to both streptomycin and tetracycline therapy and could therefore serve as reservoirs and potential donors of antibiotic resistance to other bacterial species.

## Results

### Composition of faecal microbiota in layers subjected to single-cycle therapy determined by real-time PCR

**Figure 1:**
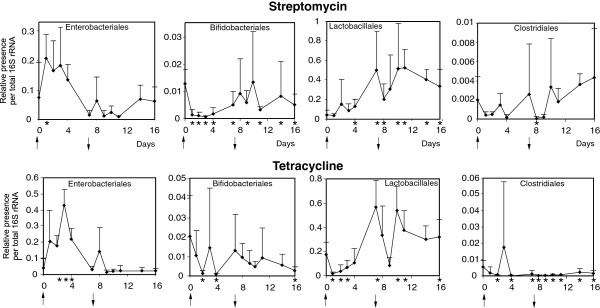
**Real-time PCR quantification of the most frequent taxa at an order level in the faeces of chickens exposed to a single dose of antibiotic therapies.** The therapy started on day 0 and lasted for 7 days as indicated by arrows. Asterisks on the X axis indicate the days in which the representation of a particular taxon differed significantly from its representation at day 0, as determined by a *t*-test (p < 0.05). Upper panels, streptomycin therapy; lower panels, tetracycline therapy.

The administration of streptomycin or tetracycline to 15-week-old layers resulted in an increased representation of *Enterobacteriales* and a decreased representation *Bifidobacteriales*, though an increased ratio of *Enterobacteriales* to total bacteria after streptomycin therapy was true for one time point only. Both these antibiotics had a similar activity on *Lactobacillales*, which increased in representation after termination of the therapy, despite a transient decrease in *Lactobacillales* immediately after the tetracycline treatment. Tetracycline also decreased representation of *Clostridiales* whereas streptomycin therapy did not influence the representation of *Clostridiales* considerably (Figure 1).

### Composition of faecal microbiota in layers subjected to repeated-cycle therapy determined by real-time PCR

**Figure 2:**
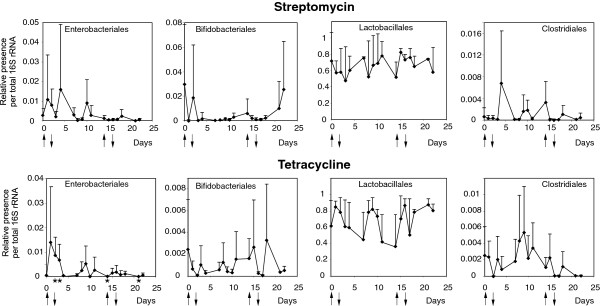
**Real-time PCR quantification of the most frequent taxa at an order level in the faeces of chickens exposed to repeated cycles of antibiotic therapy.** The therapies started on day 0, lasted for two days and were repeated on days 14 and 15, as indicated by arrows. Asterisks on the X axis indicate the days in which the representation of a particular taxon differed significantly from its representation at day 0, as determined by a *t*-test test (p < 0.05). Upper panels, streptomycin therapy; lower panels, tetracycline therapy.

Since the results in the previous experiment showed that significant changes in gut microbiota occur as early as two days after the initiation of antibiotic therapy, in the second experiment we subjected the hens only to a two-day therapy. In addition, we tested the consequences of repeated cycles of antibiotic therapy. Tetracycline therapy caused an increase in *Enterobacteriales* after the initial therapy but had no effect on *Enterobacteriales* immediately after the repeated therapy. *Lactobacillales* were resistant to both the streptomycin and tetracycline therapies, and their representation did not exhibit any clear response profile. Streptomycin and tetracycline therapies tended to decrease the representation of *Bifidobacteriales* and *Clostridiales* immediately after primary or repeated streptomycin and tetracycline administration, but soon after antibiotic withdrawal, an increase in the representation of these orders associated with a considerable day-to-day fluctuation was observed (Figure 2).

### Faecal microbiota prior to therapy determined by 454 pyrosequencing in both experiments

**Figure 3:**
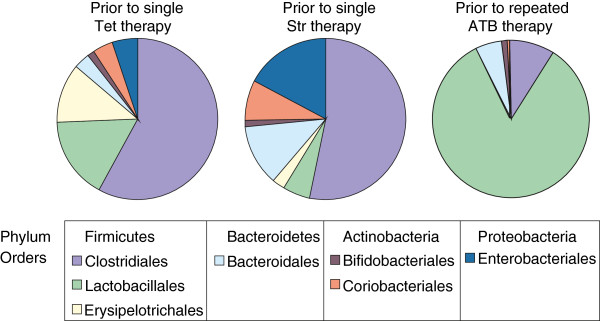
**Composition of the main orders present in chicken faecal microbiota prior to antibiotic therapy.** The sum of the appropriate orders as indicated in the figure legend provides information on the microbiota distribution at the phylum level. As in the single dose therapy experiment, the chickens were left to accommodate in two different groups, two pie charts are shown for this experiment.

To obtain a deeper insight into the changes occurring to the faecal chicken microbiota in response to the antibiotic therapy, pyrosequencing of the V3/V4 regions of the 16S rRNA genes was undertaken. In between 14,446 and 76,785 independent sequences were obtained for 11 different samples. Representatives of 13 phyla were detected at least once during the 2 experiments (Table 1 and Additional file 1: Table S1), however, the vast majority of the microbiota, over 99%, was formed by representatives of 4 phyla only; *Firmicutes*, *Bacteroidetes*, *Proteobacteria* and *Actinobacteria*. The relative representation of individual phyla in the faeces differed prior to antibiotic therapy between the 2 experiments. In the single-cycle therapy experiment, a slightly lower level of *Firmicutes* and higher levels of *Proteobacteria* and *Actinobacteria* were observed when compared with the repeated-cycle therapy experiment (Figure 3). The difference in the initial status of the chicken faecal microbiota in both the experiments was even more obvious at the order level. In the first experiment, *Clostridiales* dominated over the representatives of the remaining orders whereas the faecal microbiota of hens in the second experiment with repeated antibiotherapy was dominated by *Lactobacillales* (Figure 3).

**Table 1:** The relative representation of individual phyla (in% of total microbiota) prior to, during and after the antibiotic therapy

	**Experiment 1**				**Experiment 2**						
Day	0	0	2	2	0	2	2	14	14	16	16
phyllum	Str	Tet	Str	Tet	Str/Tet	Str	Tet	Str	Tet	Str	Tet
Bacteria. other	0.02	0.02	0.02	0.02	0.02	0.01	ND	0.09	0.46	ND	ND
Actinobacteria	7.21	4.09	0.38	0.09	1.67	8.7	0.01	0.35	1.76	0.24	0.11
Bacteroidetes	13.04	3.56	0.07	0.02	6.61	0.57	ND	2.48	11.3	0.04	ND
Cyanobacteria	0.01	ND	0.02	0.01	ND	ND	ND	ND	ND	ND	ND
Deferribacteres	ND	ND	ND	ND	0.02	ND	ND	ND	0.23	ND	ND
Firmicutes	61.83	86.60	45.07	27.81	89.88	90.3	99.88	89.84	82.94	99.22	99.8
Fusobacteria	ND	ND	ND	ND	0.77	0.12	ND	6.81	0.24	0.4	ND
Chloroflexi	ND	ND	0.003	ND	ND	ND	ND	ND	ND	ND	ND
Lentisphaerae	ND	ND	ND	ND	0.003	ND	ND	ND	ND	ND	ND
Proteobacteria	17.80	5.33	54.41	72.04	0.9	0.16	0.08	0.28	2.2	0.03	0.04
Spirochaetes	ND	ND	ND	ND	0.001	ND	ND	ND	ND	ND	ND
Synergistetes	ND	ND	ND	ND	0.1	0.07	ND	0.11	0.82	ND	ND
Tenericutes	ND	ND	ND	ND	0.001	ND	ND	ND	ND	ND	ND
TM7	0.09	0.39	0.01	ND	0.01	0.02	ND	0.01	ND	0.01	ND
OTU*	590	709	172	273	2222	433	182	896	1028	236	228
Chao1	1106	932	488	263	4592	709	263	1675	1860	490	364
OTU 14,446	478	528	131	182	869	433	164	541	799	211	165
Chao1 14,446	769	887	209	240	1791	709	290	1043	1510	422	357
Evenness	0.615	0.57	0.3	0.24	0.37	0.47	0.2	0.39	0.45	0.22	0.28
Shannon index	4.03	3.68	1.71	1.26	2.87	2.85	1.03	2.65	3.12	1.25	1.53
Total n. of reads	22,885	29,269	32,010	25,031	76,785	14,446	19,834	37,707	22,615	17,808	27,703

ND – not detected.

* OTU – number of OTUs using all reads available for each sample, Chao1 index estimates OTUs richness; OTU 14,446 and Chao1 14,446 - as above but normalised to randomly selected 14,446 reads for each sample, i.e. to the number of reads available for the sample with the lowest coverage; “Evenness” characterises how close in numbers each OTUs were present in each microbial population; Shannon index combines both species richness and evenness of their distribution within given microbial population.

### The complexity of faecal microbiota after antibiotic therapy determined by 454 pyrosequencing

Therapy with both antibiotics in both experiments reduced the complexity of gut microbiota two days after antibiotic therapy. Chao1 index estimated the total number of OTUs prior the therapy in the layers in the first experiment to 769 and 887 which decreased to 209 and 240 after two days of streptomycin and tetracycline therapy, respectively. Prior the second experiment with the repeated antibiotic therapy, Chao1 index estimated the total number of OTUs present in faeces to 1,791 which decreased to 709 and 290 after two days of streptomycin and tetracycline therapy, respectively. The interruption of antibiotic therapy for 12 days allowed for a rapid recovery of microbiota since Chao1 index increased to 1,043 and 1,510 after streptomycin and tetracycline therapy, respectively. However, the repeated antibiotic administration decreased the microbiota complexity again as the Chao1 index decreased to 422 and 357 after repeated streptomycin or tetracycline therapies, respectively (Table 1).

**Figure 4:**
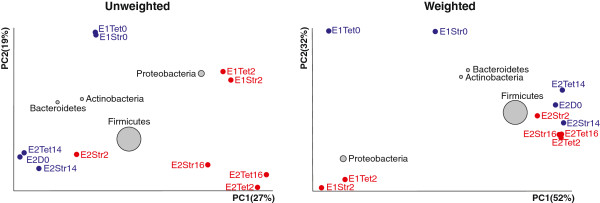
**Unweighted and weighted UniFrac PCoA of faecal microbiota from hens with and without antibiotic treatment.** Diameter of the circle for 4 major phyla present in the faeces of chickens corresponds to their average relative representation in all the samples. Sample designation - E1 or E2, experiment with single or repeated therapy; Str“n” or Tet“n”, streptomycin or tetracycline therapy where “n” indicates number of days from the beginning of the experiment. Blue designations, microbiomes of chicken prior therapy, red designations, microbiomes of chickens two days after therapy.

UniFrac β-diversity analysis followed by PCoA (principal co-ordinate analysis) indicated a clear separation between the antibiotic treated and non-treated groups using both un-weighted and weighted analysis (Figure 4). The PC1 (principal coordinate) in the un-weighted analysis, which ignores the relative representation of individual microbiota members, showed that a single factor explained 27% of all the variability among compared groups. PC1 in weighted analysis, which includes the relative representation of individual OTU into calculation, explained 52% the variability among compared groups. Displaying the microbiota representatives in biplot PCoA indicated slightly lower effect of primary streptomycin treatment in the second experiment. This analysis also clustered the representatives of phyla *Bacteroidetes* and *Actinobacteria* with the non-treated layers and *Proteobacteria* with the antibiotic-treated layers (Figure 4).

Analysis at the lower taxonomical levels showed that the therapy with both antibiotics in both the experiments always reduced the prevalence of *Bifidobacteriales*, *Bacteroidales*, *Clostridiales*, *Desulfovibrionales*, *Burkholderiales* and *Campylobacterales*. On the other hand, the orders *Enterobacteriales* and *Lactobacillales* increased in relative representation after the administration of both tetracycline and streptomycin in both experiments (Additional file 1: Table S1). When we analysed the composition of the orders in which the increase in prevalence was recorded in both experiments at the genus level, *Enterobacteriales* was comprised of representatives of the genera *Pantoea*, *Proteus*, *Citrobacter*, *Enterobacter* and *Escherichia*. However, since around 98% of all *Enterobacteriales* were formed by *Escherichia*, the increase observed for the whole order after both streptomycin and tetracycline administration was caused by the representatives of genus *Escherichia*. The order *Lactobacillales* comprised of 10 different genera, out of which the genera *Lactobacillus*, *Enterococcus*, *Paralactobacillus* and *Streptococcus* formed more than 99% of all *Lactobacillales*. However, only representatives of the genus *Enterococcus* increased in both experiments and after the therapies with both antibiotics.

### Microbiota composition along the chicken digestive tract

**Figure 5:**
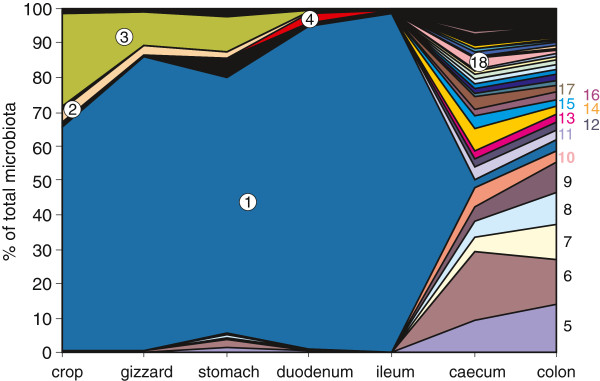
**Species distribution along the digestive tract of an adult hen.** Only the most frequent genera are identified. For complete data, see Additional file 2: Table S2. 1 – *Lactobacillus*, 2 – *Veillonella*, 3 - *Gallibacterium*, 4 – *Campylobacter*, 5 – unclassified *Lachnospiraceae*, 6 – *Bacteriodes*, 7 – *Faecalibacterium*, 8 – *Megamonas*, 9 – *Olsenella*, 10 – *Phascolarctobacterium*, 11 – *Prevotella*, 12 – *Blautia*, 13 – *Pseudoflavonifractor*, 14 – *Barnesiella*, 15 – *Desulfovibrio*, 16 - *Clostridium* XlVa, 17 – unclassified *Porphyromonadaceae*, 18 – *Alistipes*.

In the last experiment, we searched for the possible sources of microbiota which increased in representation during or after therapy. Pyrosequencing of 16S rRNA amplification products from crop, gizzard, stomach, duodenum, ileum, caecum and colon DNA resulted in between 2,332 and 25,997 independent sequences. The crop, gizzard, stomach and small intestine were mutually quite similar in composition to each other but different from the caecum and colon. The crop was dominated by *Lactobacillus* followed by *Gallibacterium* (family *Pasteurellaceae*). The less abundant genera in the crop included *Veillonella* and *Enterococcus*. *Feacalibacterium* and *Bacteroides* were detected in the stomach although these genera were otherwise characteristic for the caecum and colon. Both parts of the small intestine were dominated by *Lactobacillus* species. The diversity of microbiota considerably increased in the caecum and colon (Chao1 OTU estimates predicted 342 different OTUs in the crop, 1,028 in the gizzard, 733 in the stomach, 1,223 in the duodenum, 169 in the ileum, 1,821 in the caecum and 4,647 in the colon) with different strict anaerobes forming the majority of microbiota members (Figure 5 and Additional file 2: Table S2).

## Discussion

In this study we characterised chicken faecal microbiota and changes induced by antibiotic therapy by real time PCR and pyrosequencing of 16S rRNA amplification products. The results from the real time PCR must be considered with increased care as it was rather difficult to design primer pairs specific for orders as diverse as *Clostridiales*. This can be clearly seen if data from Figure 1 and 2 are compared with Figure 3. If Y axis scaling in Figures 1 and 2 is multiplied by a factor of 100, the same percentage out of total microbiota as in Figure 3 can be deduced. Such comparison shows that real time PCR detection of *Enterobacteriales* and *Lactobacillales* provided similar data as pyrosequencing but *Bifidobacteriales* and *Clostridiales* were highly underestimated by the real time PCR. Being aware of this, we used the real time PCR mainly to characterise time-dependent changes in microbiota composition, which should be less affected by the relaxed specificity of the 2 real time PCRs, and also to obtain some information on hen-to-hen variation.

The composition of faecal microbiota prior to the antibiotic therapy was similar to that reported in previous studies in chickens i.e. *Firmicutes* dominating, followed by *Bacteroidetes* and *Proteobacteria*[1,4,7]. In the 15-week layers, there was a higher proportion of *Proteobacteria* at the expense of *Firmicutes* when compared with the 46-week old hens, which may correlate with known gradual colonisation patterns in young animals [8,13] but may also be a consequence of the 3-week adaptation to a new, clean experimental animal house prior to the first experiment.

The changes in microbiota composition induced by the antibiotic therapy were rapid and quite dramatic as in between 55 (streptomycin therapy in the first experiment) to 94% (first tetracycline administration during the second experiment) of OTU disappeared or decreased below the detection limit within 48 after antibiotic administration. Only representatives of the genera *Enterococcus* and *Escherichia* increased in response to the therapy with both antibiotics in both experiments, despite the fact that layers of different age and with different microbiota composition were used in the two experiments. However, one has to be reminded that the increases reported in this study may not necessarily correlate with the increase in total bacterial counts of the appropriate taxon. If streptomycin or tetracycline inactivated certain groups of bacteria but left the others unaffected, the latter will increase in proportion but not in actual numbers. Since the chickens or hens were kept under the same conditions, all the birds were provided the same feed and for the duration of the experiment, they were kept in animal house with air conditioning and strict hygienic regime minimising the external sources of microbiota to technical minimum, we did not include a control non-treated group. We believe that such extensive changes in gut microbiota upon antibiotic administration were direct consequences of the therapy and not of random fluctuation in gut microbiota composition.

Interestingly, the origin of the representatives of *Enterococcus* and *Escherichia* could be quite different. Although we performed the microbiota characterisation along the digestive tract only in a single set of samples, representatives of *Enteroccocus* were present only in the proximal parts of the digestive tract (crop, gizzard, stomach) while representatives of *Escherichia* were found in caecum or colon. On the other hand, sources of *Lactobacillus* which increased in faeces in the single cycle therapy experiment could originate from the crop till the jejunum. Not surprisingly we observed the increase in *Lactobacillus* prevalence in faeces only in the single-dose therapy experiment where a low representation of *Lactobacillus* was observed prior to the therapy, leaving a space for *Lactobacillus* increase after the therapy withdrawal. This could not happen in the experiment with repeated therapy as in this case, the *Lactobacillus* representation was quite high prior to antibiotic therapy not allowing for an additional increase. The restoration of microbiota complexity after therapy withdrawal was nearly as rapid as the changes immediately after the therapies. Twelve days after the withdrawal, the estimated number of OTUs increased and PCoA analysis clustered microbiomes of such layers with that of the non-treated layers. One of the potential reservoirs for such a rapid microbiota restoration could be found in the stomach, since in this organ we found certain microbiota members which were otherwise common to caecum, colon or faeces.

## Conclusions

Although the experiments described in this study have been performed on a limited number of hens and in pooled samples, we observed that the changes in microbiota composition induced by the antibiotic therapy were rapid and quite dramatic and only representatives of the genera *Enterococcus* and *Escherichia* increased in response to the therapy with both antibiotics in both experiments. Interestingly, the restoration of microbiota complexity after therapy withdrawal was nearly as rapid as the changes immediately after the therapies. The stomach can be understood as one of the possible reservoirs for such a rapid microbiota restoration since certain microbiota members, which were otherwise common to caecum, colon or faeces, were found also in this organ.

## Methods

### Experimental animals

Female Lohmann Brown layers, obtained from a commercial producer with no history of antibiotic use, were used in this study. The handling of animals in the study was performed in accordance with current Czech legislation. The specific experiments were approved by the Ethics Committee of the Veterinary Research Institute followed by the Committee for Animal Welfare of the Ministry of Agriculture of the Czech Republic.

### Single-cycle therapy

Twelve-week-old layers were transferred from a farm and allowed to adapt in the experimental animal house for 3 weeks prior to the antibiotic therapy. During the adaptation period, the layers were divided into two groups kept at separate rooms, each group consisting of 5 layers. Daily water consumption of the groups of 5 birds was determined and this information was used to provide layers with the antibiotics in the drinking water at such a concentration that the daily uptake was equivalent to 60 mg of tetracycline or 15 mg of streptomycin per kg of body weight, respectively. The drinking water with antibiotics was administered successively for 7 days and faecal samples were individually collected from each layer. The first sampling was performed when the layers were 15-week old, just prior to the antibiotic administration (day 0), followed by sampling on days 1, 2, 3, 4, 7, 8, 9, 10, 11, 14 and 16, when the experiment was terminated.

### Repeated-cycle therapy

The aim of this experiment was to characterise the changes in the faecal microbiota of hens subjected to repeated cycles of antibiotic therapy. Two groups of five, 46-week-old hens were brought to the institute, housed in separate rooms, subjected to antibiotic therapy without any adaptation with the same antibiotics and at the same dosage as in single-cycle experiment. Furthermore, since in the first experiment we found that the greatest changes in gut microbiota were observed as early as two days after the antibiotic therapy, we treated the hens with the antibiotics for two days only, let them recover for 12 days without antibiotic administration, and on day 14, the therapy was repeated for additional two days. Faecal samples were individually collected on days 0, 1, 2, 3, 4, 7, 8, 9, 10, 11, 14, 15, 16, 17, 18, 21 and 22, when the experiment was terminated.

### Characterisation of microbiota along the digestive tract

Three adult 46-week-old hens were sacrificed and the contents of the crop, gizzard, stomach, duodenum, ileum, caecum and colon were taken for DNA purification. The composition of microbiota was determined in all the samples from all 3 birds by real-time PCR (see below), and the samples from the hen exhibiting the median values in most of the real time PCRs were subjected to pyrosequencing of the 16S rRNA amplification products (see below).

### DNA purification and taxon-specific real-time PCR

DNA was extracted from the faeces by QIAamp DNA Stool Mini Kit according to the manufacturer’s instructions (Qiagen) and stored at −20°C until use. Taxon-specific primers were designed from the variable regions of 16S rRNA genes with PRIMROSE software [14] and the specificity of the primers was tested by RDP ProbeMatch program. Two primer pairs specific for the conservative regions of 16S rRNA genes (domain *Bacteria* universal primer pairs) served to determine the total bacterial DNA present in the samples (Table 2). Real-time PCR was carried out using QuantiTect SYBR Green PCR Kit (Qiagen) in a LightCycler LC480 thermocycler (Roche). The PCR was initiated with a hot start for 15 min at 95°C followed by 45 cycles of 20 sec at 95°C, 30 sec at 60°C and 30 sec at 72°C. Melting temperatures were determined after PCR to verify the correctness of each PCR product. After PCR, the Ct values of the genes of interest were subtracted from an average Ct value of amplifications performed with universal primer pairs for the domain *Bacteria* (ΔCt). The relative amount of each taxon in the total bacterial population was finally calculated as 2^-ΔCt^[15,16].

**Table 2:** List of primers used in this study

**Primer**	**Target**	**Sequence 5 **^**′**^**- 3**^**′**^	**Reference**
16S_Bifido-F	*Bifidobacteriales*	GGTGTGAAAGTCCATCG	this study
16S_Bifido-R	*Bifidobacteriales*	ACCGGGAATTCCAGTCT	this study
16S_Clost-F	*Clostridiales*	GCGTTATCCGGATTTAC	this study
16S_Clost-R	*Clostridiales*	ACACCTAGTATTCATCG	this study
16S_Entero-F	*Enterobacteriales*	STGAGACAGGTGCTGCA	this study
16S_Entero-R	*Enterobacteriales*	AAAGGATAAGGGTTGCG	this study
16S_Lacto-F	*Lactobacillales*	CTTGAGTGCAGAAGAGG	this study
16S_Lacto-R	*Lactobacillales*	CACTGGTGTTCTTCCAT	this study
16S_univ-1 F	all bacteria	GTGSTGCAYGGYTGTCGTCA	[17]
16S_univ-1R	all bacteria	ACGTCRTCCMCACCTTCCTC	[17]
16S_univ-2 F	all bacteria	GAGGAAGGIGIGGAIGACGT	[13]
16S_univ-2R	all bacteria	AGICCCGIGAACGTATTCAC	[13]

### Pyrosequencing

Equal amounts of faecal DNA originating from the layers of the same treatment group and time were pooled prior to PCR and used as a template in PCR with forward primer 5^′^CGTATCGCCTCCCTCGCGCCATCAG-MID-*GGAGGCAGCAGTRRGGAAT*- 3^′^ and reverse primer 5^′^CTATGCGCCTTGCCAGCCCGCTCAG-MID-*CTACCRGGGTATCTAATCC*-3^′^. The underlined sequences were required at different steps of amplicon pyrosequencing. The sequences in italics are specific to the conserved sequences of bacterial 16S rRNA genes allowing amplification of the V3/V4 hypervariable region [18]. MID represents different 10 bp sequences which enable simultaneous sequencing and re-identification of DNA originating from different samples. After PCR, the amplification products were loaded onto a 1.2% agarose gel, separated by gel electrophoresis and gel-purified using a QIAEX II Gel Extraction Kit (Qiagen). Pyrosequencing was performed using a GS Junior 454 sequencer and GS Junior Titanium sequencing chemistry exactly according to the manufacturer’s instructions (Roche). In one sequencing run, the amplification products from 4 samples were mixed and analysed.

### Sequence analysis

The FASTA and qual files generated as an output of pyrosequencing were uploaded into Qiime software [19]. Quality trimming criteria included no mismatch in the bar code sequences and maximally 1 mismatch in the primer sequences. In the next step, chimeric sequences were predicted and excluded from the analysis. The obtained sequences were then classified with RDP Seqmatch with an OTU (operational taxonomic units) discrimination level set up to 97%. Diversity analyses (Chao1 richness, Evenness estimation and Shannon index) on OTU clusters were performed using all sequences available for each sample. Finally, UniFrac analysis [20] followed by principal coordinate analysis and Biplot data visualisation was used to characterise diversity in the microbial populations tested.

### Statistics

Data from real-time PCR are presented as average ± SD. The comparison of taxon representation at a particular day to the representation on day 0, i.e. prior to antibiotic therapy, was evaluated by a *t*-test using SPSS v.14 statistical software.

## Competing interests

The authors state that they do not have and financial or personal conflicts that could inappropriately bias their work.

## Authors’ contributions

PV performed the pyrosequencing, analysed the data and helped to draft the manuscript. MF and HJ purified the DNA, designed real time PCR primers and performed the real time PCR. FS and HH were responsible for the animal experiments and sample collection. VB performed the statistical analysis. IR participated in the design of the study, data analysis and helped to draft the manuscript. All authors read and approved the final manuscript.

## Supplementary Material

Additional file 1: Table S1 List of all reads and OTUs identified after antibiotic treatment in both experiments of this study. Click here for file

Additional file 2: Table S2 List of all reads and OTUs identified along digestive tract of adult hen. Click here for file

## References

[B1] Zhu XY Zhong T Pandya Y Joerger RD 16S rRNA-based analysis of microbiota from the cecum of broiler chickens Appl Environ Microbiol 2002 68 124 137 10.1128/AEM.68.1.124-137.2002 11772618 PMC126585

[B2] Knarreborg A Simon MA Engberg RM Jensen BB Tannock GW Effects of dietary fat source and subtherapeutic levels of antibiotic on the bacterial community in the ileum of broiler chickens at various ages Appl Environ Microbiol 2002 68 5918 5924 10.1128/AEM.68.12.5918-5924.2002 12450811 PMC134372

[B3] Lu J Idris U Harmon B Hofacre C Maurer JJ Lee MD Diversity and succession of the intestinal bacterial community of the maturing broiler chicken Appl Environ Microbiol 2003 69 6816 6824 10.1128/AEM.69.11.6816-6824.2003 14602645 PMC262306

[B4] Nordentoft S Molbak L Bjerrum L De Vylder J Van Immerseel F Pedersen K The influence of the cage system and colonisation of Salmonella Enteritidis on the microbial gut flora of laying hens studied by T-RFLP and 454 pyrosequencing BMC Microbiol 2011 11 187 10.1186/1471-2180-11-187 21859465 PMC3188488

[B5] Bokkers EA de Boer IJ Economic, ecological, and social performance of conventional and organic broiler production in the Netherlands Br Poult Sci 2009 50 546 557 10.1080/00071660903140999 19904633

[B6] Amit-Romach E Sklan D Uni Z Microflora ecology of the chicken intestine using 16S ribosomal DNA primers Poult Sci 2004 83 1093 1098 15285498 10.1093/ps/83.7.1093

[B7] Qu A Brulc JM Wilson MK Law BF Theoret JR Joens LA Konkel ME Angly F Dinsdale EA Edwards RA Comparative metagenomics reveals host specific metavirulomes and horizontal gene transfer elements in the chicken cecum microbiome PLoS One 2008 3 e2945 10.1371/journal.pone.0002945 18698407 PMC2492807

[B8] Crhanova M Hradecka H Faldynova M Matulova M Havlickova H Sisak F Rychlik I Immune response of chicken gut to natural colonization by gut microflora and to Salmonella enterica serovar Enteritidis infection Infect Immun 2011 79 2755 2763 10.1128/IAI.01375-10 21555397 PMC3191970

[B9] Dethlefsen L Huse S Sogin ML Relman DA The pervasive effects of an antibiotic on the human gut microbiota, as revealed by deep 16S rRNA sequencing PLoS Biol 2008 6 e280 10.1371/journal.pbio.0060280 19018661 PMC2586385

[B10] Antonopoulos DA Huse SM Morrison HG Schmidt TM Sogin ML Young VB Reproducible community dynamics of the gastrointestinal microbiota following antibiotic perturbation Infect Immun 2009 77 2367 2375 10.1128/IAI.01520-08 19307217 PMC2687343

[B11] Jernberg C Lofmark S Edlund C Jansson JK Long-term impacts of antibiotic exposure on the human intestinal microbiota Microbiology 2010 156 3216 3223 10.1099/mic.0.040618-0 20705661

[B12] Robinson CJ Young VB Antibiotic administration alters the community structure of the gastrointestinal micobiota Gut Microbes 2010 1 279 284 10.4161/gmic.1.4.12614 20953272 PMC2954510

[B13] Tseng CP Cheng JC Tseng CC Wang C Chen YL Chiu DT Liao HC Chang SS Broad-range ribosomal RNA real-time PCR after removal of DNA from reagents: melting profiles for clinically important bacteria Clin Chem 2003 49 306 309 10.1373/49.2.306 12560356

[B14] Ashelford KE Weightman AJ Fry JC PRIMROSE: a computer program for generating and estimating the phylogenetic range of 16S rRNA oligonucleotide probes and primers in conjunction with the RDP-II database Nucleic Acids Res 2002 30 3481 9 10.1093/nar/gkf450 12140334 PMC137075

[B15] Juricova H Videnska P Lukac M Faldynova M Babak V Havlickova H Sisak F Rychlik I Influence of Salmonella enterica serovar Enteritidis infection on the development of cecum microbiota in newly hatched chicks Appl Environ Microbiol 2013 79 745 7 10.1128/AEM.02628-12 23144133 PMC3553771

[B16] Matulova M Rajova J Vlasatikova L Volf J Stepanova H Havlickova H Sisak F Rychlik I Characterization of chicken spleen transcriptome after infection with Salmonella enterica serovar Enteritidis PLoS One 2012 7 e48101 10.1371/journal.pone.0048101 23094107 PMC3477135

[B17] Maeda H Fujimoto C Haruki Y Maeda T Kokeguchi S Petelin M Arai H Tanimoto I Nishimura F Takashiba S Quantitative real-time PCR using TaqMan and SYBR Green for Actinobacillus actinomycetemcomitans, Porphyromonas gingivalis, Prevotella intermedia, tetQ gene and total bacteria FEMS Immunol Med Microbiol 2003 39 81 86 10.1016/S0928-8244(03)00224-4 14557000

[B18] Nossa CW Oberdorf WE Yang L Aas JA Paster BJ Desantis TZ Brodie EL Malamud D Poles MA Pei Z Design of 16S rRNA gene primers for 454 pyrosequencing of the human foregut microbiome World J Gastroenterol 2010 16 4135 4144 10.3748/wjg.v16.i33.4135 20806429 PMC2932916

[B19] Caporaso JG Kuczynski J Stombaugh J Bittinger K Bushman FD Costello EK Fierer N Pena AG Goodrich JK Gordon JI QIIME allows analysis of high-throughput community sequencing data Nat Methods 2010 7 335 336 10.1038/nmeth.f.303 20383131 PMC3156573

[B20] Lozupone C Knight R UniFrac: a new phylogenetic method for comparing microbial communities Appl Environ Microbiol 2005 71 8228 8235 10.1128/AEM.71.12.8228-8235.2005 16332807 PMC1317376

